# Elevated expression of CST1 promotes breast cancer progression and predicts a poor prognosis

**DOI:** 10.1007/s00109-017-1537-1

**Published:** 2017-05-18

**Authors:** Da-nian Dai, Yan Li, Bo Chen, Yong Du, Shi-bing Li, Shi-xun Lu, Zhi-ping Zhao, Ai-jun Zhou, Ning Xue, Tian-liang Xia, Mu-sheng Zeng, Qian Zhong, Wei-dong Wei

**Affiliations:** 1 State Key Laboratory of Oncology in South China, Collaborative Innovation Center for Cancer Medicine, Sun Yat-Sen University Cancer Center, Guangzhou, Guangdong China; 2 Department of Breast Oncology, Sun Yat-Sen University Cancer Center, 651 East Dongfeng Road, Guangzhou, 510060 China; 3 Department of Pathology, Sun Yat-Sen University Cancer Center, Guangzhou, China; 4 Institute of Hepatopancreatobiliary Surgery, Southwest Hospital, Third Military Medical University, Chongqing, China

**Keywords:** CST1, Breast cancer, Prognostic biomarker

## Abstract

**Abstract:**

Cystatin SN (CST1) belongs to the type 2 cystatin (CST) superfamily, which restricts the proteolytic activities of cysteine proteases. CST1 has been recently considered to be involved in the development of several human cancers. However, the prognostic significance and function of CST1 in breast cancer remains unknown. In the current study, we found that CST1 was generally upregulated in breast cancer at both mRNA and protein level. Furthermore, overall survival (OS) and disease-free survival (DFS) in the low CST1 expression subgroup were significantly superior to the high CST1 expression subgroup (OS, *p* < 0.001; DFS, *p* < 0.001), which indicated that CST1 expression level was closely correlated to the survival risk of these patients. Univariate and multivariate analyses demonstrated that CST1 expression was an independent prognostic factor, the same as ER status and nodal status. Next, CST1 overexpression promoted breast cancer cell proliferation, clonogenicity, migration, and invasion abilities. By contrast, knockdown of CST1 attenuated these malignant characteristics in breast cancer cells. Collectively, our study indicates that CST1 cannot only serve as a significant prognostic indicator but also as a potential therapeutic target for breast cancer.

**Key messages:**

- High CST1 expression is negatively correlated with survival of breast cancer patients.
- CST1 promotes cell proliferation, clone formation, and metastasis in breast cancer cells.
- CST1 is a novel potential prognostic biomarker and therapeutic target for breast cancer.

**Electronic supplementary material:**

The online version of this article (doi:10.1007/s00109-017-1537-1) contains supplementary material, which is available to authorized users.

## Background

The malignant neoplasm with the highest incidence in women is breast cancer all over the world and one out of 18 women develops breast cancer during the period from birth to age 79 years [1]. Breast cancer mortality has been declining since 1991, which suggests a benefit from the combination of early detection and more effective comprehensive treatment of local disease with surgery, radiation therapy, endocrine therapy, and systemic treatment with chemotherapy and so on [2]. However, prevention and therapy of breast cancer still remains a major public health concern.

At the present stage, surgical resection continues to be the classic therapy for breast cancer patients. Even though patients with the same molecular subtype of breast cancer receive the same therapy, they have different outcomes [3, 4]. Hence, better predictive factors of prognosis are required to evaluate the outcome of therapy and provide guidance for individualized treatment.

Cysteine proteases, also known as thiol proteases, are enzymes with the activity of protein degradation which is associated with a diversity of diseases and facilitates the development and progression of cancer cells [5–7]. For neoplastic diseases, one of the important roles of cysteine cathepsins is confirmed by accumulating evidence in the progression, invasion, and metastasis of solid tumors and they seem to influence the response of tumor cells to ionizing radiation and cytostatic agents [8–10]. Meanwhile, there is also experimental evidence highlighting that lysosomal cathepsins, in particular cathepsin B, D, and L, may also trigger activation of multiple signaling pathways, which may also result in cell death. In this case, these proteolytic enzymes act as tumor suppressors [11, 12]. The CST1 gene encodes a secretory protein called CST1, which belongs to the type 2 cystatin (CST) superfamily, including CST1, CST2, CST3, CST4, and CST5. These members restrict the proteolytic activities of cysteine proteases [13]. Numerous studies have implied that cystatins play important roles in tumor invasion and metastasis. For example, the salivary cystatins CST1, CST2, CST3, and CST4 were associated with both local invasion at the early stage and remote metastasis in colorectal cancer [14, 15]. In addition, CST5 has tumor suppressor activity that may contribute to the antitumoral action of 1 alpha, 25(OH)2D3 in colon cancer [16]. It has been proven that the inter-restricted relationship between cystatins and cysteine proteases influences a variety of pathological processes, including seasonal allergic rhinitis, chronic kidney disease, and tumor invasion and metastasis, which are correlative with regulation of the proteolytic system [17–22]. Cystatins may also act through mechanisms which are independent from their cathepsin inhibiting activity (e.g., modulation of immune system, and/or apoptosis, and/or cell senescence or interactions with growth factors) [23–26]. Furthermore, previous studies report that CST1 was upregulated in cancerous lesions of gastric cancer tissue, which suggests its important role in the regulation of proteolysis system and its effect on gastric tumorigenesis through T cell factor-mediated proliferative signaling [27]. These reports imply that CST1 may contribute to the process of carcinogenesis and tumor progression. Nonetheless, the effect and mechanisms of CST1 in breast cancer has not yet been elucidated. Therefore, this study was aimed to investigate the expression and the role of CST1 in breast cancer.

In the current study, we analyzed microarray data from the TCGA database and revealed that CST1 was upregulated in breast cancer compared with normal tissues. We then compared CST1 messenger RNA (mRNA) and protein expression in breast cancer and normal tissue using real-time PCR (RT-PCR), western blotting, and immunohistochemistry (IHC). In addition, we also explored the function of the CST1 protein in breast cancer cells through gain or loss of function assays. Furthermore, the primary goal of this study is to assess the predictive impact of CST1 expression on recurrence, metastasis, and survival in patients with surgically resected breast cancer. Taken together, our findings demonstrate that CST1 was highly expressed in breast cancer and suggests that CST1 might be a potential therapeutic target.

## Materials and methods

### Microarray

The TCGA datasets of breast cancer which contain 231 samples of invasive breast carcinoma and normal breast were utilized for gene expression profiling in the public Oncomine website (https://www.oncomine.org). All the original data were further processed with MultiExperiment Viewer 4.9 to get the heat map.

### Patients and tissue specimens

This study was agreed to be conducted by the Institutional Review Board and Human Ethics Committee of Sun Yat-Sen University Cancer Center through the review. Prior to surgery, all patients signed the written consent for their samples to be used in scientific research.

Ten breast cancer tissues and their adjacent paired non-tumorous mammary tissues were collected from Sun Yat-sen University Cancer Center (SYSUCC), Guangzhou, China. These specimens were immediately stored at −80 °C until further processed.

For IHC analysis, 244 paraffin-embedded tumor tissues were obtained from female breast cancer patients with definite diagnosis by two pathologists in Sun Yat-Sen University Cancer Center, China, during the period from October 2002 to December 2009. IHC of ER, PR, and HER-2 status were performed in the Pathology Department of Sun Yat-Sen University Cancer Center.

The eligibility criteria of the current study were summarized as follows: (1) all the patients recruited in this study were histologically and clinically diagnosed, (2) none of the patients had distant metastasis and received neoadjuvant/adjuvant treatments and radiotherapy before mammectomy, (3) serious complications (i.e., cancer cachexia syndrome, heart disease, diabetes) or other malignant diseases were not diagnosed, (4) detailed clinicopathological data (e.g., age, histological type, lymph nodes status, tumor size, stage, ER status, PR status, HER-2 status, local relapse, and distant metastatic relapse) for all these patients were available and reviewed (Table S1). The histological type, which was reclassified according to the WHO classification and tumor stage, was based on the TNM staging system (American Joint Committee on Cancer Classification, 7th edition, http://www.cancerstaging.org).

Follow-up data (e.g., recurrence, metastasis, survival status, death, and the causes of death) after surgery was updated by a review of the records and telephone calls. All patients were followed up every 3–5 months during the first 5 years and every year thereafter. Overall survival (OS) was computed from the date of surgery to the date of death or last follow-up. Disease-free survival (DFS) was defined as from the date of surgery to the date of relapse, metastasis, or last follow-up. All patients’ survival statuses were confirmed in February 2015.

### Cell culture

The immortalized human breast epithelial cell line (76N-tert) was cultured in keratinocyte serum-free medium (Invitrogen, Carlsbad, CA, USA). The breast cancer cell lines (MDA-MB-468, BT-474, MCF-7, MDA-MB-435, SKBr-3, MDA-MB-415, BT-549, MDA-MB-231) were cultured in Dulbecco’s modified Eagle’s medium (DMEM; Gibco, Carlsbad, CA, USA) supplemented with 10% fetal bovine serum (FBS; Gibco) in a humidified 5% CO_2_ incubator at 37 °C. 76N-tert was isolated from a reduction mammoplasty specimen and immortalized by ectopic overexpression of hTERT [28, 29]. Luminal cell lines (BT-474, MCF-7, SKBr-3, MDA-MB-415) have been associated with a more differentiated, noninvasive phenotype. Basal-like cell lines (BT-549, MDA-MB-468, MDA-MB-435, MDA-MB-231) have been associated with a more invasive phenotype [30].

### Quantitative RT-PCR analysis

Total RNA was isolated from tissue specimens by using the TRIzol reagent (Invitrogen) according to the manufacturer’s instructions. RNA concentration and quality were determined with NanoDrop spectrophotometer (ND-1000, Thermo Scientific, Massachusetts, USA). Complementary DNA (cDNA) was synthesized using 2 μg of the total RNA according to a reverse transcriptase kit (Invitrogen). qRT-PCR with the Power SYBR Green qPCR SuperMix-UDG (Invitrogen) was used to measure the mRNA level of the target genes on an ABIPrism-7500 Sequence Detector System (ABI, Applied Biosystems, Carlsbad, USA). β-actin was used as an internal control. Relative expression of the CST1 was normalized to the expression of β-actin, which yielded a 2–∆ Ct value. The primer sequences were as follows:

- CST1 sense: 5′-CCTGTGCCTTCCATGAACAGCC-3′,
- CST1 antisense: 5′-GGGTGGTGGCTGGTGCCAATG-3′,
- β-actin sense: 5′-CGCGAGAAGATGACCCAGAT-3′,
- β-actin antisense: 5′-GGGCATACCCCTCGTAGATG-3′.

All reactions were run in triplicate.

### Western blotting analysis

Proteins from breast cancer tissues or cell lines were extracted using RIPA lysis buffer with a proteinase inhibitor. The protein concentration in the lysates was measured using the BCA Protein Assay Kit (Thermo Scientific, USA) and 20 μg of the total protein, mixed with 1 × SDS loading buffer, was loaded per lane. The proteins in the lysates were separated by 10% SDS-polyacrylamide gel electrophoresis (PAGE) and transferred to polyvinylidene difluoride (PVDF) membranes (Pall, Port Washington, USA). To block nonspecific binding, the membranes were incubated at room temperature for 1 h with 5% skim milk powder. The PVDF membranes were then incubated with anti-CST1 (diluted 1:200; Proteintech, USA), anti-α-tubulin (diluted 1:3000; Santa Cruz Biotechnology, Texas, USA), anti-β-actin (diluted 1:3000; Sigma-Aldrich, A5441, St. Louis, USA), anti-c-Myc (diluted 1:1000; Cell Signaling, #9402, Danvers, MA, USA), anti-cyclin E (diluted 1:1000; Upstate (Millipore), #05–363, Inc., Lake Placid, NY), anti-fibronectin (diluted 1:250; BD Biosciences, #610077, Franklin Lakes, New Jersey), and anti-E-cadherin (diluted 1:1000; BD Biosciences, #610181, Franklin Lakes, New Jersey) at 4 °C overnight. The blots were then treated with an HRP-conjugated secondary antibody (diluted 1:3000; Pierce, Rockford, IL, USA) and signal intensities were quantified by integrated density measurements on ImageJ software [31].

### Immunohistochemical analysis

The paraffin-embedded breast cancer samples were cut into 4 μm sections. Then the sections were baked for 2 h at 60 °C, deparaffinized in xylenes, and rehydrated using a series of graded alcohols. Then, the tissue slides were incubated with 3% hydrogen peroxide for 15 min to exhaust endogenous peroxidase activity. Then, the sections were boiled in EDTA antigen retrieval solution (pH = 8.0) for 2 min and 30 s in an electric pressure cooker antigen retrieval. After that, the sections were incubated with anti-CST1 antibody at a dilution of 1:50 (NBP1-55995, Novus, Littleton, USA) overnight at 4 °C in a moist chamber and a secondary antibody for 30 min at 37 °C on the next day. Subsequently, the sections were incubated with streptavidin-horseradish peroxidase complex and developed with 3-diaminobenzidine tetrahydrochloride (DAB) and counterstained with Mayer’s hematoxylin to stain nucleus. Finally, the sections were dehydrated and mounted. As a negative control, the primary antibody was replaced by normal rabbit serum. Each section was independently evaluated by two pathologists, who were blinded to the clinical status of the patients. The biomarker was evaluated according to staining intensity scored as (0, negative staining; 1, weak staining; 2, moderate staining; 3, strong staining) and the extent of staining scored as the percentage of positive cells (1, 0∼25%; 2, 26∼50%; 3, 51∼75%; 4, 76∼100%) [32]. The final quantitation of each staining was obtained by multiplying the two scores. CST1 expression was classified as high expression if the score was higher than the median score of 1.5; if the score was 1.5 or less, the case was classified as low expression.

### Establishment of stable CST1 over-expressing cell lines

CST1 cDNA was amplified by PCR and inserted into pMSCV vectors. The primers of CST1:

BamH I-sense: 5#-CGGGATCCATGGCCCAGTATCTGAGTAC-3# and Xhol I-anti sense: 5#-CCGCTCGAGCTACTTATCGTCGTCATCCTTGTAATC-3#. The vector or pMSCV-CST1 plasmid was transfected into 293T cells along with the retroviral packaging vector PIK. After transfection, the supernatants were harvested and used to infect BT-549 and MDA-MB-415, and the stably transfected cells were selected with puromycin and validated by western blotting analysis.

### Transfection with siRNA against CST1

The small interfering RNAs (siRNAs) targeting the mRNA of human CST1 (Gene ID: 1469 NM_001898.2) were denoted as siCST1-#1 (GGTGAAATCCAGGTGTCAA) and siCST1-#2 (CAGAAGGTCCCTGGTGAAA). The negative control (NC) RNA duplex for the siRNA was indicated as NC and was not homologous to any human genome sequences. BT-474 (3 × 105) and MDA-MB-415 cells (1.5 × 105) were seeded into 6-well plates, incubated for 18 h, and then transfected with 20 nM of the RNA duplex and 5 μl of Lipofectamine RNAiMAX (Invitrogen, Grand Island, NY, USA) according to the manufacturer’s instructions. The cells were harvested for further experiments after 48 or 72 h, including western blotting, MTT, colony formation assay, and transwell assays.

### MTT assay

The MTT (3-(4,5-dimethylthiazol-2-yl)-2,5-diphenyltetrazolium) assay was used to measure the viability of the breast cancer cells. The transfection group and control group respectively were seeded onto a 96-well plate at a density of 1000 cells per well and the cells were cultured with DMEM containing 10% FBS. At the indicated time points, 20 μl of 5 mg/mL MTT was added to each well, and the cells were incubated for 4 h at 37 °C, then 200 μl dimethyl sulfoxide (DMSO) was added per well to the cultured cells to dissolve the crystals. The absorbance was measured at 490 nm with a SpectraMax M5 (Molecular Devices, Sunnyvale, USA) plate reader.

### Colony formation assay

After transfection with the specific siRNAs, BT-474 and MDA-MB-468 cells (2000 cells per plate) were respectively seeded in a 35-mm plate and cultured for 14 days in a humidified 5% CO_2_ incubator at 37 °C. For stable CST1 over-expressing cell lines (BT-549 and MDA-MB-415), 1000 cells were seeded per plate and cultured for 10 days. Colonies were fixed in methanol for 10 min and then stained with 0.5% crystal violet for 20 min. All visible colonies were quantified by manual counting. At least three independent experiments were performed for each assay.

### Transwell migration and invasion assays

2 × 10^4^ cancer cells (BT-549 and MDA-MB-415) or 10 × 10^4^ cancer cells (BT-474 and MDA-MB-468) suspended in medium without FBS were seeded on each upper chamber and the bottom chambers were filled with 600 μl medium containing 10% FBS. After culturing the cells for 24 h, noninvasive cells on the upper surface of filters were completely removed with a cotton swab. Migrated cells adhered to the lower surface of filter and were rinsed with phosphate-buffered saline (PBS), fixed with methanol, stained with 1% crystal violet, and counted the number of cells at five random ×100 fields per well [33]. Cell counts are expressed as the mean number of cells per field of view. 3 × 10^4^ cancer cells (BT-549 and MDA-MB-415) or 15 × 10^4^ cancer cells (BT-474 and MDA-MB-468) were seeded for the invasion assay, with the commercially available transwell invasion chambers (Corning, NY, USA, 354480) which had been pre-coated with a uniform layer of dried basement membrane matrix solution. The rest of the procedure was the same as the migration assay. At least three independent experiments were performed for each assay.

### Statistical analysis

Data were analyzed by the SPSS standard version 20.0 (SPSS, Chicago, USA). The ROC curve was generated by the SPSS standard version 20.0 and the maximum Youden index (sensitivity + specificity—1) was defined as cutoff value [34–36]. The chi-square test was used to analyze the correlation of CST1 status with clinicopathological features. The Kaplan–Meier method was used to estimate OS, and the Cox multivariate proportional hazards regression model was used to determine the independent factors that influence survival and recurrence based on the investigated variables. Differences between subgroups were analyzed with the two-tailed Mann–Whitney test. *p* values less than 0.05 were considered as statistically significant.

## Results

### Upregulation of CST1 in breast cancer

**Fig. 1:**
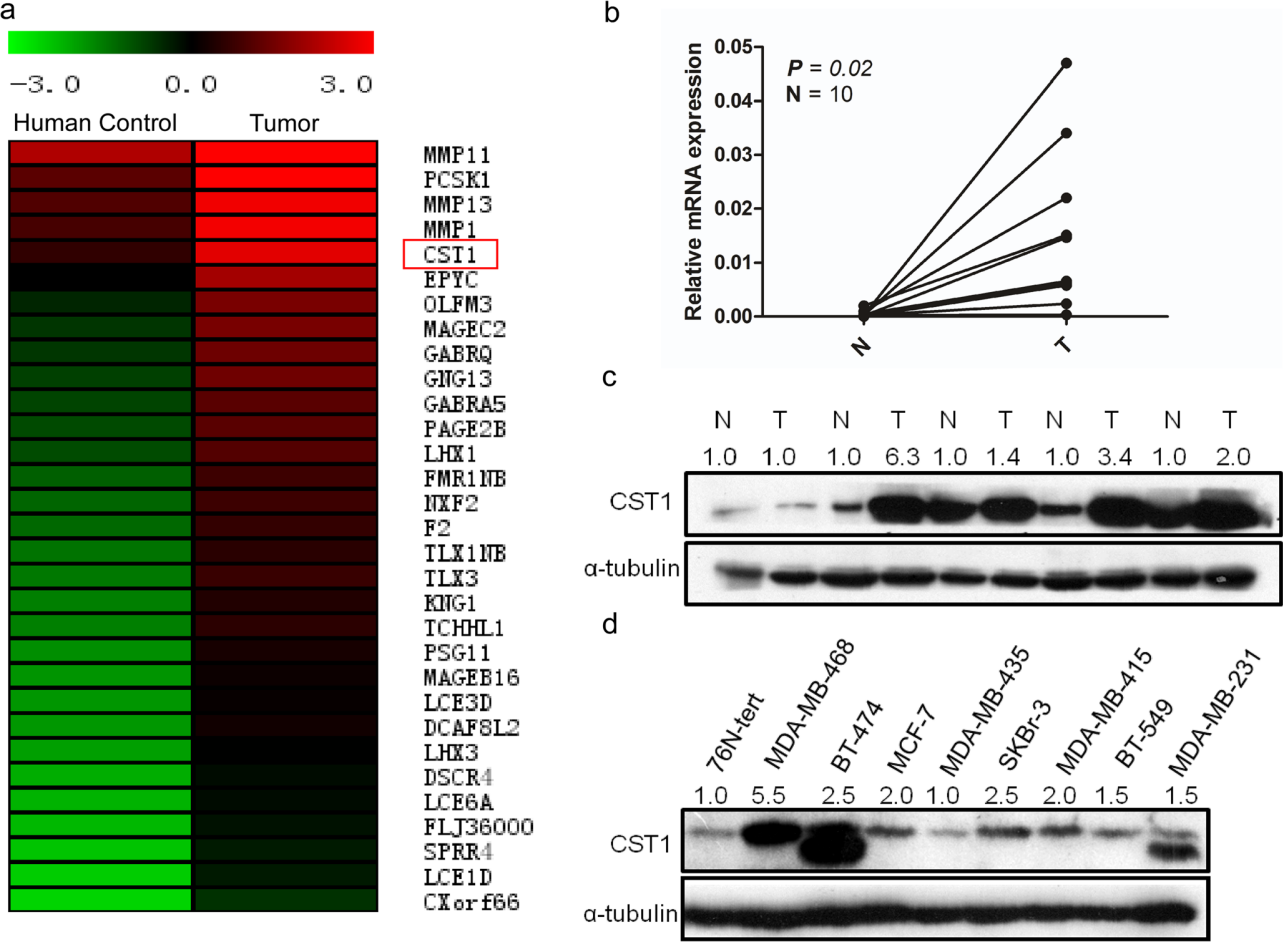
Overexpression of CST1 in breast cancer. **a** CST1 was significantly upregulated in breast cancer tissues compared with normal breast tissues according to a microarray from TCGA database of Oncomine website. CST1 is marked by a *red box*. **b**, **c** CST1 mRNA and protein levels in 10 or 5 pairs of matched breast cancer and non-tumor tissues, respectively. **d** The CST1 protein level in a panel of breast cancer cell lines including MDA-MB-468, BT-474, MCF-7, MDA-MB-435, SKRr-3, MDA-MB-415, BT-549, MDA-MB-231 compared with the immortalized human breast epithelial cell 76N-tert. mRNA levels are presented as means ± SD and normalized to the housekeeping gene β-actin by qRT-PCR. *N* matched noncancerous tissue, *T* tumor tissue

To investigate new potential predictive factors of prognosis in breast cancer, the RNA expression profiles of breast cancer tissues and normal tissues were compared through microarray data derived from TCGA datasets (http://tcga-data.nci.nih.gov/tcga/), which were analyzed on Oncomine websites. CST1 was upregulated almost fourfold in breast cancer tissues compared with normal breast tissues (Fig. 1a). To further confirm this result, RT-PCR and western blot were performed in ten paired breast cancer tissues and adjacent normal breast tissues. Consistent with the microarray data, CST1 was upregulated both at mRNA level (Fig. 1b) and protein level (Fig. 1c) in breast cancer tissues compared with corresponding adjacent normal breast tissues. Furthermore, the expression of CST1 was relatively higher in breast cancer cell lines compared with the immortalized human breast epithelial cell line 76N-tert (Fig. 1d).

### CST1 expression in breast cancer tissues and normal breast tissues

**Fig. 2:**
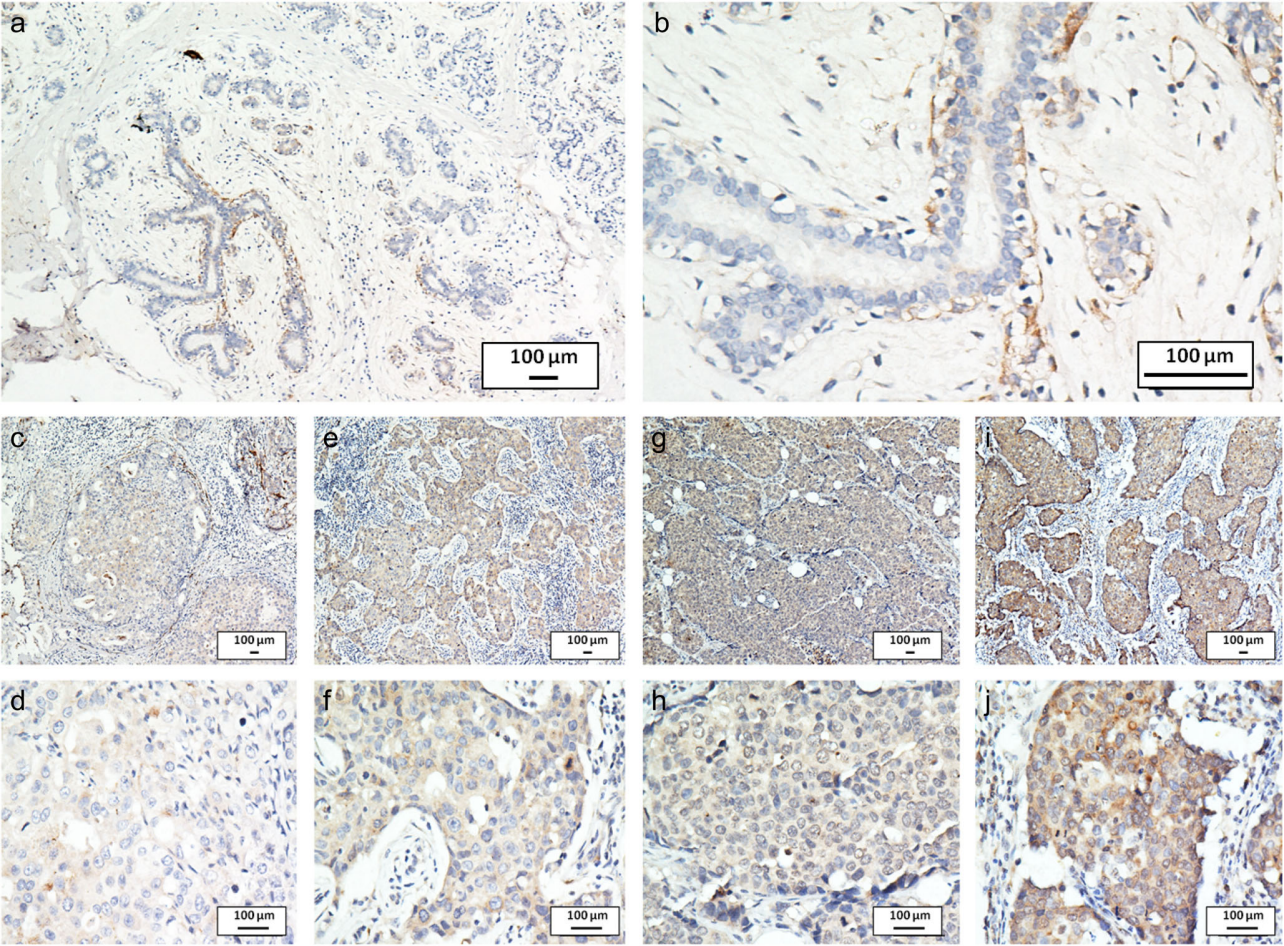
CST1 expression analyzed by immunohistochemical staining. Negative CST1 staining in normal breast ductal epithelium (negative control) **a** ×100, **b** ×400, negative staining of CST1 in breast cancer tissue **c** ×100, **d** ×400, weak staining of CST1 in cytoplasm **e** ×100, **f** ×400, moderate staining of CST1 in cytoplasm **g** ×100, **h** ×400, and strong staining of CST1 in cytoplasm **i** ×100, **j** ×400

To examine the expression and localization of CST1 in breast cancer tissues, immunohistochemistry analysis was applied in 244 paraffin-embedded specimens. CST1 expression was significantly upregulated in breast cancer tissues compared with the adjacent normal tissues (Fig. 2a, b). As shown in Fig. 2c–j, the expression of CST1 was localized to the cytoplasm. To determine an optimal cutoff value to differentiate low CST1 expression, a ROC curve was used on the basis of the results of IHC staining analysis (Fig. S1). The ROC curve for clinical stage possessed the smallest distance (0.0, 1.0), manifesting that CST1 expression has the greatest prognostic ability (maximum sensitivity and specificity) for clinical stage. Therefore, an IRS score of 1.5 was chosen as a cutoff value for low CST1 expression and 193 (79.1%) of the 244 tumors exhibited high CST1 expression, whereas 51 (20.9%) exhibited low CST1 expression.

### Association between CST1 expression and clinicopathological variables

To investigate whether CST1 is linked to clinicopathological variables in breast cancer, we analyzed the correlation between CST1 expression and clinicopathological variables in 244 breast cancer samples. As summarized in Table S1, the chi-square test showed that CST1 expression was significantly correlated with menarche age (*p* = 0.017), tumor size (*p* = 0.005), HER-2 status (*p* = 0.045), and histological grade (*p* < 0.001). However, the expression of CST1 had no significant correlation with age, menopause, pathological LN infiltrated, tumor location, ER status, PR status, triple-negative breast cancer, pathological tumor status, and pathological TNM staging (all *p* > 0.05).

### Significant association between high expression of CST1 and poor prognosis in breast cancer patients

**Fig. 3:**
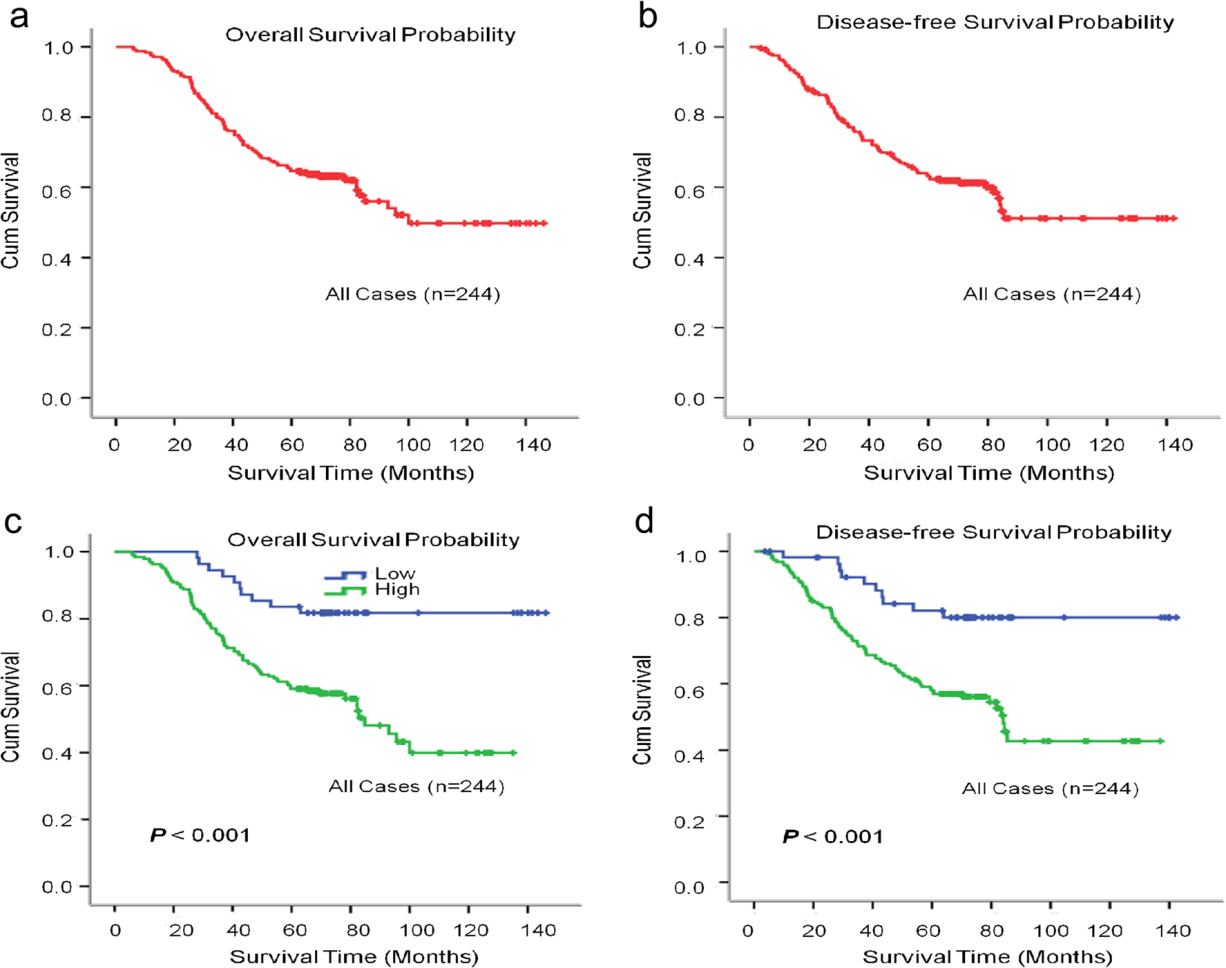
Kaplan–Meier estimates of the probability of survival for patients with breast cancer. **a**, **b** Five-year overall survival (OS) and disease-free survival (DFS) rates of 244 breast cancer patient were 65.3 and 62.5% respectively; **c**, **d** High CST1 expression level was significantly correlated to OS (*p* < 0.001) and DFS (*p* = 0.001) in all breast cancer patients

To explore the CST1 status on OS and DFS in breast cancer patients, the correlation between CST1 expression and the survival of patients with breast cancer was analyzed by Kaplan–Meier methodology (log-rank test). Among the 244 patients in this study, 98 patients were deceased and 146 were alive at the final clinical follow-up, and the median observation period was 65 months (with a range of 6 to 146 months). The 5-year overall survival and disease-free survival rate were 65.3 and 62.5%, respectively, in the total cohort (Fig. 3a, b). Patients with high CST1 expression demonstrated a significant worse OS and DFS than those with low CST1 expression (Fig. 3c, d). The mean OS was 134.5 and 83.8 months in the low and high CST1 expression subgroups, respectively (Table S2). Consistently, the mean DFS of patients with high CST1 expression (83.1 months) was shorter than that of patients with low CST1 expression (127.7 months) (Table S2).

**Fig. 4:**
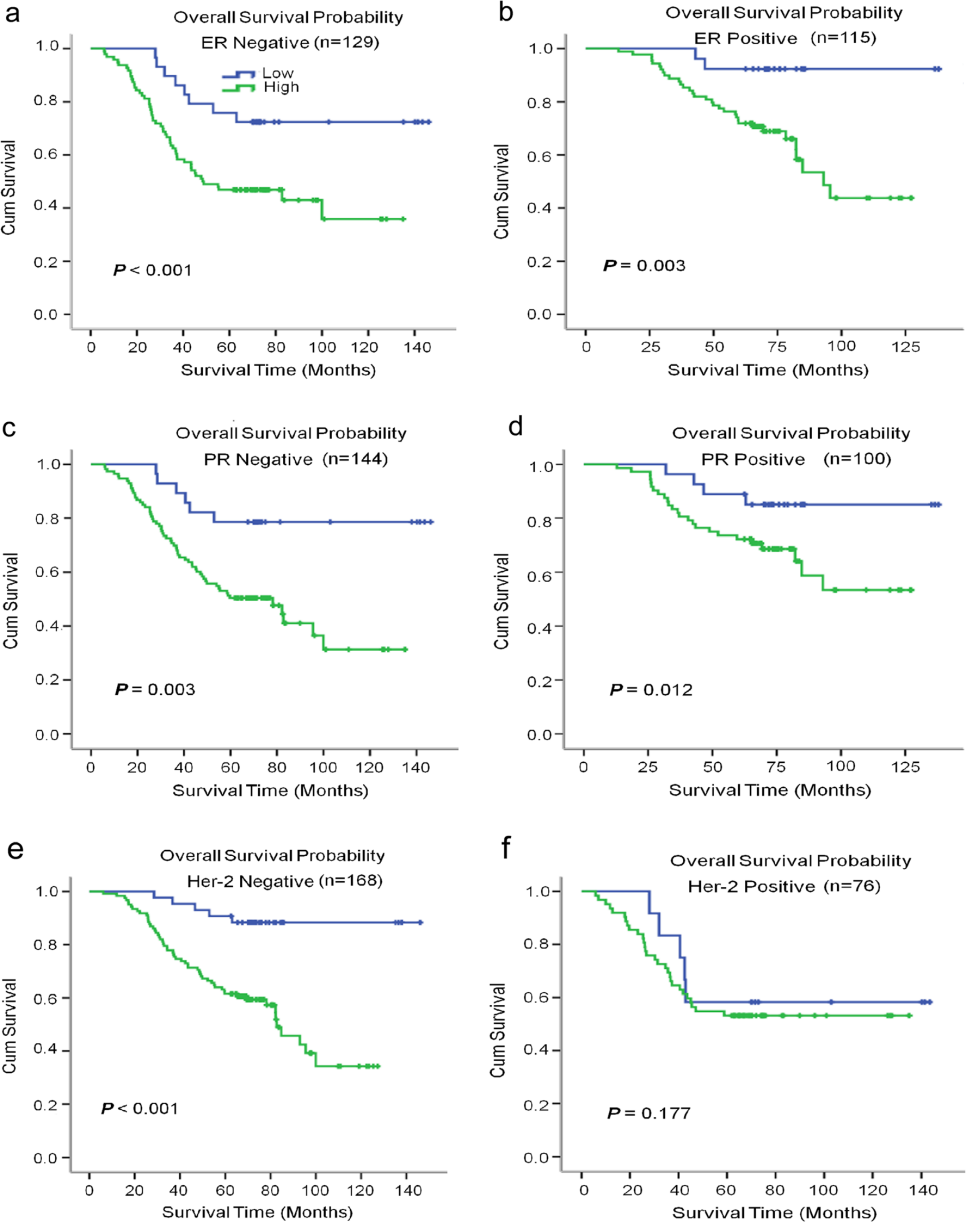
Kaplan–Meier estimates of the probability of survival for patients with breast cancer according to the receptor expression in classification. All patients were stratified according to ER status, PR status, and HER-2 status. **a**, **b** The overall survival (OS) rate of breast cancer patients in different ER status. **c**, **d** The overall survival (OS) rate of breast cancer patients in different PR status. **e**, **f** The overall survival (OS) rate of breast cancer patients in different HER-2 status

**Fig. 5:**
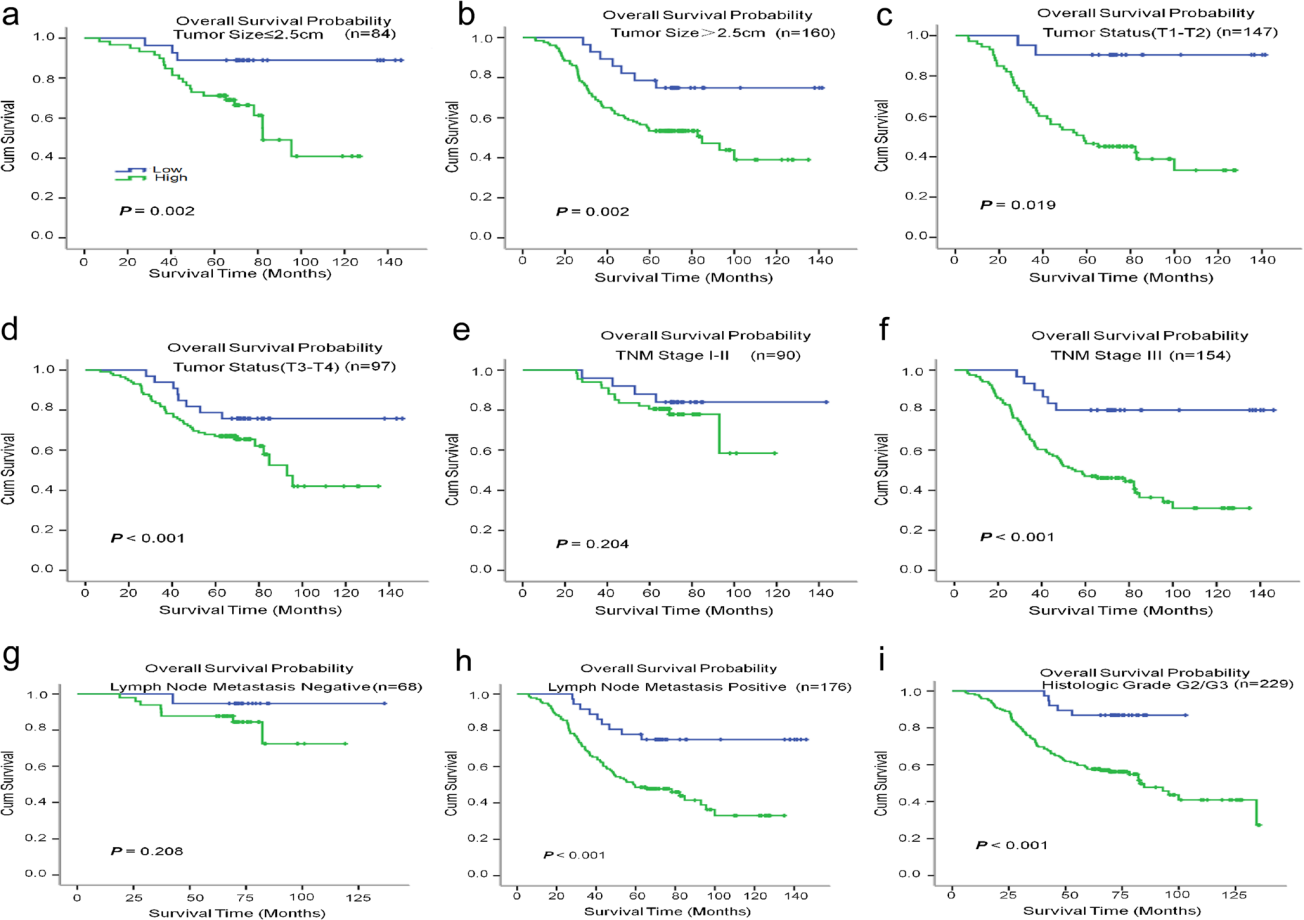
Kaplan–Meier estimates of the probability of survival for patients with breast cancer according to the general pathological indicators in classification. All patients were stratified according to tumor size, tumor differentiation, TNM classification, lymph node metastasis status, and histological grade. **a**, **b** The overall survival (OS) rate of breast cancer patients with different tumor sizes. **c, d** The overall survival (OS) rate of breast cancer patients in different tumor status. **e**, **f** The overall survival (OS) rate of breast cancer patients in different TNM classification. **g**, **h** The overall survival (OS) rate of breast cancer patients with different lymph node metastasis status. **i** The overall survival (OS) rate of breast cancer patients in histological grade

To further investigate the prognostic value of CST1 in different subgroups, patients were stratified according to the three main receptor expression groups, including ER status (Fig. 4a, b), PR status (Fig. 4c, d), and HER-2 status (Fig. 4e, f), and the common pathological indicators (Fig. 5a–i), including tumor size, tumor differentiation, TNM stage, lymph node metastasis status, and histological grade. The high expression of CST1 remained its prognostic value in predicting worse OS and DFS in most of abovementioned subgroups such as tumor size, tumor differentiation, lymph node metastasis positive, ER/PR status, HER2 negative, TNM staging III, and histologic grade (Table S2).

Additionally, we performed univariate analysis using a Cox proportional hazard model to validate the effectiveness of each independent factor on OS and DFS. As exhibited in Tables S3 and S4, the univariate survival analysis showed that tumor size (OS, HR = 1.680, *p* = 0.023; DFS, HR = 1.619, *p* = 0.035), histological grade (OS, HR = 2.544, *p* < 0.001; DFS, HR = 2.380, *p* < 0.001), nodal status (OS, HR = 4.544, *p* < 0.001; DFS, HR = 4.803, *p* < 0.001), TNM staging (OS, HR = 2.826, *p* < 0.001; DFS, HR = 2.931, *p* < 0.001), ER status (OS, HR = 0.514, *p* = 0.002; DFS, HR = 0.485, *p* = 0.001), PR status (OS, HR = 0.536, *p* = 0.004; DFS, HR = 0.512, *p* = 0.002), and CST1 expression level (OS, HR = 5.611, *p* < 0.001; DFS, HR = 5.083, *p* < 0.001) were independent predictors for DFS and OS. However, the multivariate survival analysis model revealed predominantly independent predictors for OS and DFS were nodal status (OS, HR = 3.287, *p* = 0.002; DFS, HR = 3.332, *p* = 0.002), histological grade (OS, HR = 1.908, *p* = 0.002; DFS, HR = 1.828, *p* = 0.003), and CST1 expression level (OS, HR = 3.390, *p* = 0.006; DFS, HR = 2.791, *p* = 0.013).

### Overexpression of CST1 promotes proliferation and colony formation of breast cancer cells

**Fig. 6:**
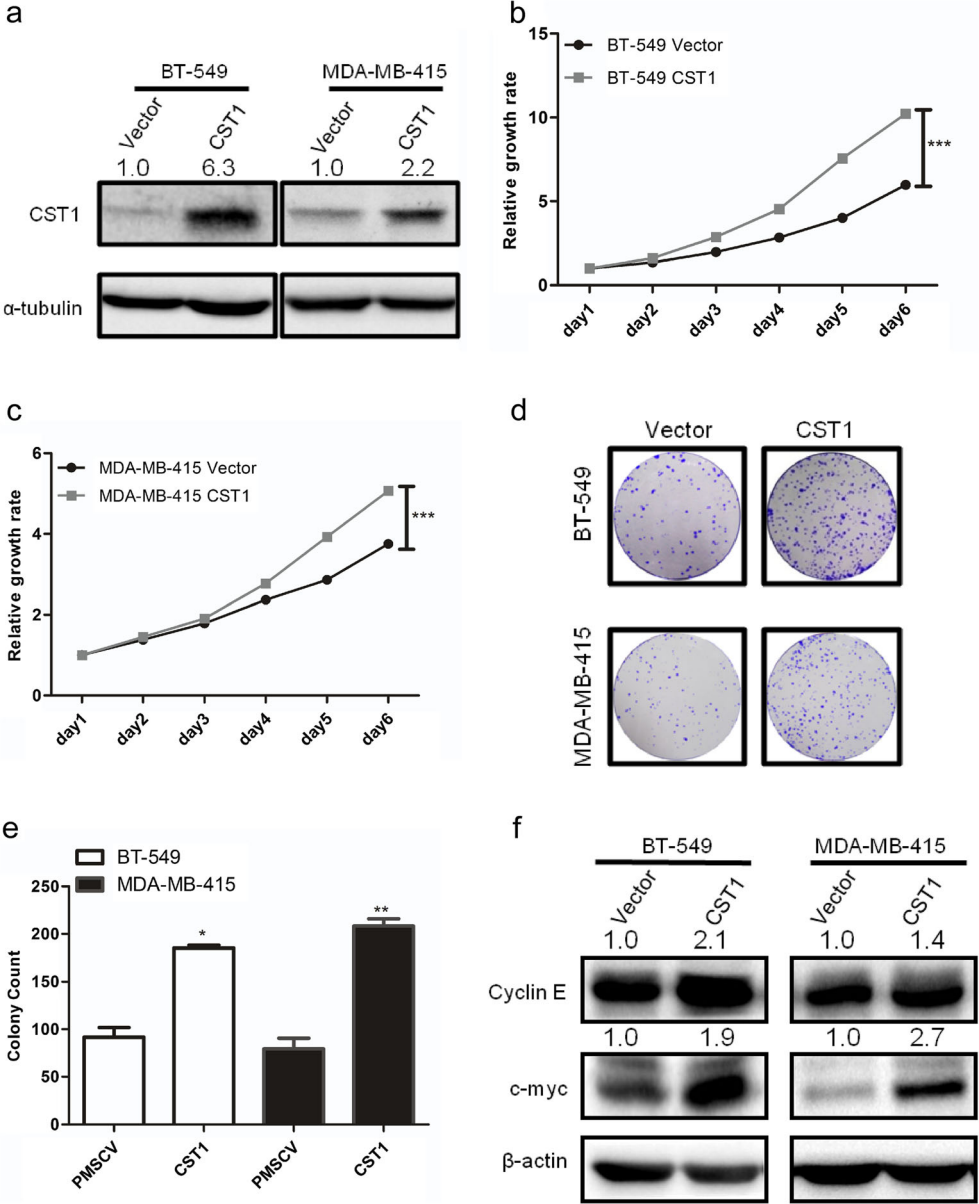
Overexpression of CST1 promotes proliferation in breast cancer cells. **a** Expression levels of CST1 protein after stable transfection with vector or CST1-expressing plasmids were detected by western blotting analysis. **b** Cell proliferation after CST1 overexpression in BT-549 cells was measured by MTT assay. **c** Cell proliferation after CST1 overexpression in MDA-MB-415 cells was measured by MTT assay. **d** Cell colony formation was determined in BT-549 and MDA-MB-415 cells expressing vector or CST1. **e** The number of colonies of BT-549 and MDA-MB-415 cells harboring vector or CST1. **f** The representative pictures of proliferation-related proteins after overexpression of CST1 in BT-549 and MDA-MB-415 cells, as determined by western blotting. Results are expressed as means ± SD (*error bars*). *p < 0.05, **p < 0.01, and ***p < 0.001 compared to vector

To investigate the function of CST1 in breast cancer, we exogenously overexpressed CST1 in two breast cancer cell lines, BT-549 and MDA-MB-415 (Fig. 6a). We found that CST1 promotes cell growth of BT-549 and MDA-MB-415 cells (Fig. 6b, c) examined by MTT assay, suggesting CST1-enhanced cell proliferation. Besides, overexpression of CST1 significantly enhanced the number of colonies of BT-549 and MDA-MB-415 cells, suggesting that CST1 induced the colony-forming ability in breast cancer (Fig. 6d, e). In addition, a remarkable increase of the c-Myc and Cyclin E proteins was detected in CST1 overexpressed cells (Fig. 6f). These results indicated that CST1 markedly enhanced breast cancer cell proliferation and clonogenicity in vitro.

### Knockdown of CST1-attenuated proliferation and colony formation of breast cancer cells

**Fig. 7:**
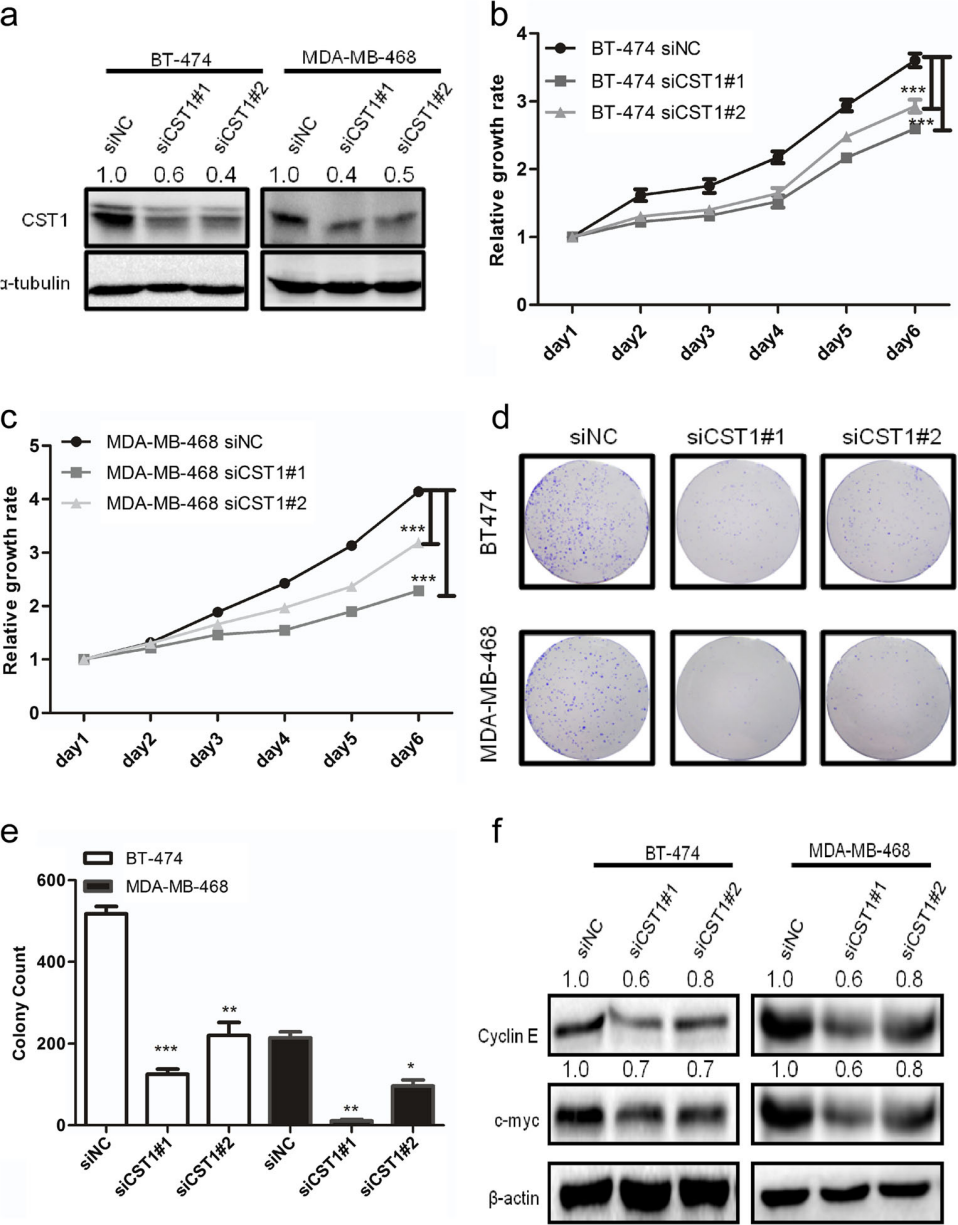
Knockdown of CST1 suppresses proliferation in breast cancer cells. **a** Knockdown of CST1 resulted in reduced CST1 protein expression in BT-474 and MDA-MB-468 cell lines. **b** Cell proliferation after CST1 knockdown in BT-474 cells was measured by MTT assay. **c** Cell proliferation after CST1 knockdown in MDA-MB-468 cells was measured by MTT assay. **d** Cell colony formation was determined in BT-474 and MDA-MB-468 cells transfected with NC- or CST1-targeting siRNAs. **e** The number of colonies after knockdown of CST1 in BT-474 and MDA-MB-468 cells. **f** The representative pictures of proliferation-related proteins after knockdown of CST1, as determined by western blotting. Results are expressed as means ± SD (*error bars*). *p < 0.05, **p < 0.01, and ***p < 0.001 compared to siNC

To explore the function of endogenous CST1 in breast cancer, we knocked down CST1 expression in BT-474 and MDA-MB-468 cells by transiently transfecting two independent siRNAs against CST1 (Fig. 7a). Next, the MTT assay was performed to examine whether reducing the expression of CST1 could inhibit cell proliferation. The results displayed that CST1 knockdown markedly suppressed cancer cell viability (Fig. 7b, c). Moreover, we also noticed that inhibition of CST1 in BT-474 or MDA-MB-468 cells dramatically caused sparser colonies compared with the negative control (Fig. 7d, e). Similarly, a distinct decrease of the c-Myc and Cyclin E proteins was observed in CST1-silenced cells (Fig. 7f). All of these results suggest that the CST1 could exhibit oncogenic functions in breast cancer.

### CST1 promotes migration and invasion of breast cancer cells

**Fig. 8:**
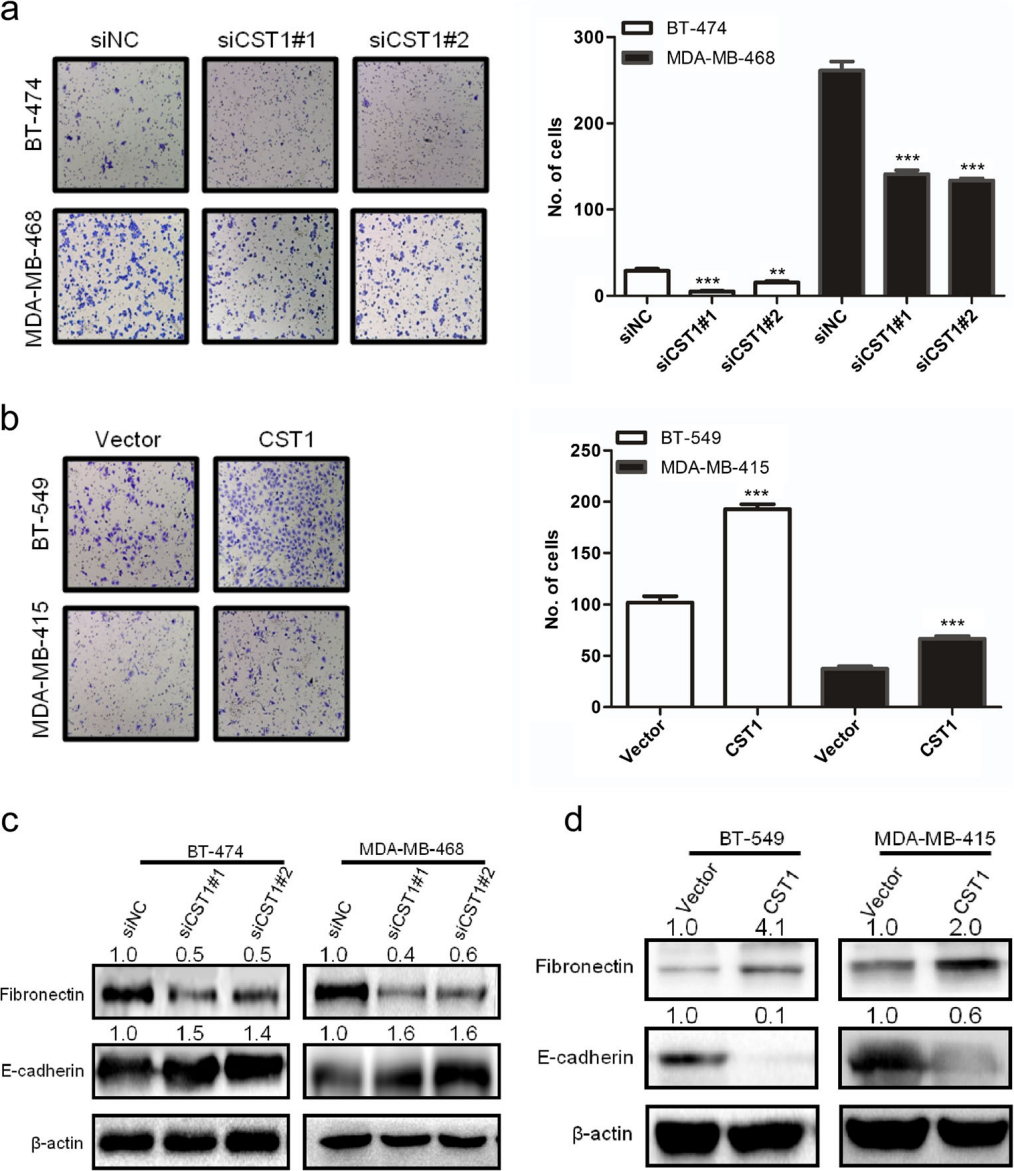
CST1 promotes migration in breast cancer cells. **a** The representative pictures and quantification of migration in BT-474 and MDA-MB-468 cells transfected with NC- or CST1-targeting (KD) siRNAs by transwell migration assay after the knockdown of CST1. **b** The representative pictures and quantification of cell migration in BT-549 and MDA-MB-415 cells harboring vector or pMSCV-CST1 plasmid by transwell migration assay. **c** The representative pictures of metastasis-related proteins after the knockdown of CST1, as determined by western blotting. **d** The representative pictures of metastasis-related proteins after the overexpression of CST1, as determined by western blotting. Migrating cells were identified using light microscope (×100). Results are expressed as means ± SD (*error bars*) from five viewing fields. *p < 0.05, **p < 0.01, and ***p < 0.001 compared to siNC or vector

**Fig. 9:**
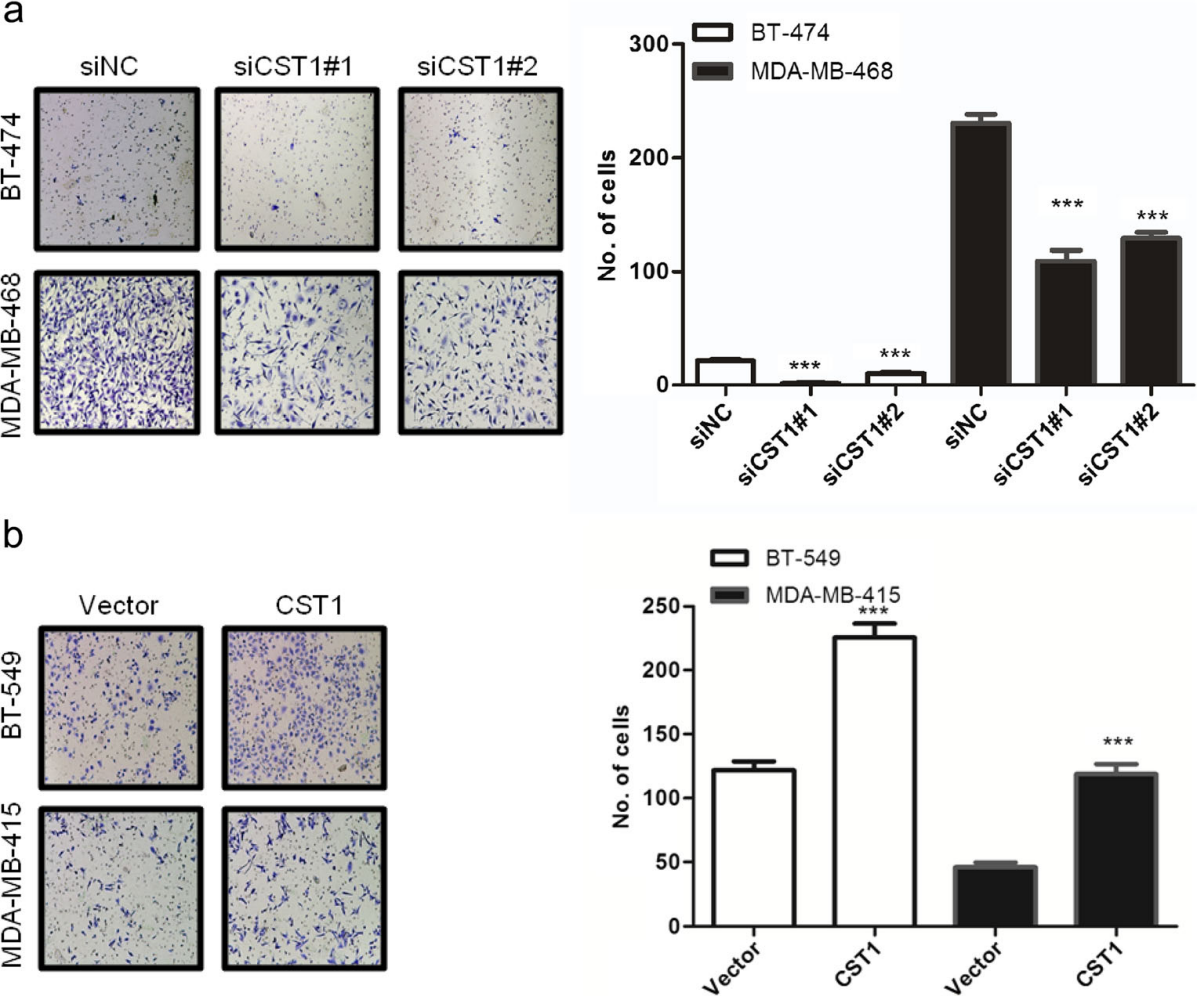
CST1 promotes invasion of breast cancer cells. **a** The representative pictures and quantification of invasion of BT-474 and MDA-MB-468 cells transfected with NC- or CST1-targeting (KD) siRNAs by transwell invasion assay after the knockdown of CST1. **b** The representative pictures and quantification of cell invasion in BT-549 and MDA-MB-415 cells harboring vector or pMSCV-CST1 plasmid by transwell invasion assay. The invasive cells were identified using light microscope (×100). Results are expressed as means ± SD (*error bars*) from five viewing fields. *p < 0.05, **p < 0.01, and ***p < 0.001 compared to siNC or vector

As shown in the clinical data, CST1 expression correlated with metastasis of breast cancer. Thus, we investigated the effect of CST1 on migration and invasion in breast cancer. Different transwell chambers were employed to determine cell migration or invasion. To eliminate the interference caused by the proliferative action, all cells were starved in serum-free medium for 24 h before this test. As expected, knockdown of CST1 expression in BT-474 and MDA-MB-468 cells significantly inhibited cell migration capability (Fig. 8a) and invasion capability (Fig. 9a). On the contrary, overexpression of CST1 strongly stimulated cell migration and invasion in BT-549 and MDA-MB-415 cells (Figs. 8b and 9b). To explore the effects of CST1 on epithelial-mesenchymal transition (EMT), mesenchymal and epithelial markers, including fibronectin and E-cadherin, were examined. Interestingly, the above described biological phenomena were also in accordance with the changes of metastasis-related proteins analyzed by western blotting (Fig. 8c, d). Collectively, these data indicate that CST1 could promote migration and invasion in breast cancer cells, at least in part, through the enhancement of EMT.

## Discussion

Breast cancer is a heterogeneous cancer with a very high incidence in women. Seeking for valuable biomarkers for breast cancer prognostic assessment has an important influence for the treatment of breast cancer. Over the years, many potential biomarkers have been revealed to be of diagnostic value in breast cancer, such as serum angiopoietin-2 [37], annexin A1 [38], and miR-22 [39]. As far as we know, our study is the first scientific analysis to assess the expression, clinicopathologic significance, prognostic value, and functional effect of CST1 in breast cancer.

In the present study, CST1 was dramatically increased in breast cancer cell lines, and most of breast cancer tissue samples compared with adjacent non-cancer controls at both mRNA and protein levels. In addition, IHC staining of breast cancer samples revealed that CST1 is predominantly located in the cytoplasm and highly expressed in breast cancer tissues compared with adjacent non-malignant controls. Moreover, ectopic introduction of CST1 resulted in elevation of proliferation, colony formation, migration, and invasion. In contrast, knockdown of CST1 reduced the above characteristics. Collectively, these data indicated that overexpression of CST1 was coincident with tumor progression, which suggests CST1 as a tumor promoter in breast cancer. In accordance with this, the Kaplan–Meier survival analysis demonstrated that the OS and DFS of breast cancer patients with high CST1 expression were worse than those with low CST1 expression. High CST1 expression was also reported by previous studies to be linked to poor survival in colorectal cancer [40], pancreatic cancer [41], and non-small cell lung cancer patients [42], which was consistent with our results. Interestingly, the prognostic effect of CST1 is especially strong in stratified survival analysis of breast cancer, according to the factors attributed to worse outcome. Therefore, high CST1 expression could identify a subgroup of breast cancer patients with worse prognosis. This implies that elevated CST1 in breast cancer could serve as a feasible prognostic factor for patients with breast cancer in risk groups.

Strikingly, high CST1 expression could also predict poor overall survival of breast cancer patients and different subgroups with ER status, PR status, HER-2 status (negative), tumor size, tumor status, lymph node metastasis status (positive), TNM stage (III), or histological stage. Generally, most of patients with hormone-receptors (ER/PR) positive were sensitive to hormone therapy. However, as shown in Fig. 4, those with high CST1 levels displayed greatly reduced survival rates, which probably implied resistance to conventional medication therapy. Even among terminal cancer patients (Fig. 5f), the distinction of survival rates with different CST1 status was obvious. These results showed CST1 expression may serve as a supplementary factor to direct patient treatment together with molecular classification. Therefore, a better therapeutic effect could be achieved if patients received more aggressive treatment or targeted CST1 treatment according to their CST1 expression level. Finally, the current results indicate that upregulation of CST1 is a risk factor for tumor recurrence and metastasis, and its expression associated with clinicopathological parameters may act as an independent predictor for patient survival and a therapeutic target in breast cancer patients.

However, the underlying molecular mechanisms of how CST1 is involved in tumorigenesis remain unclear. In our present study, CST1 may promote proliferation and metastasis of breast cancer by regulating cell cycle and inducing EMT process. Interestingly, published literature has shown that type 2 cystatin superfamily has about >70% sequence similarity. Likewise, at the protein level, S-type cystatins share a high extent of amino acid similarity (∼88%) [43–45], which implies that the same subtype of cystatins may have the similar mechanism. The regulation of the stability of cystatins as well as counter-balancing related to cysteine protease and cystatin complexes may affect the prognosis of cancer patients. In addition, previous studies confirmed the previous hypothesis that the inhibitory effect of CST3 on cathepsin B activity was reduced by 50% through the effects of CST1, which may contribute to the dissociation of the CST3–CTSB complex, thus leading to tumorigenesis at a certain extent [46, 47]. Taken together, all of the above described findings offer certain evidence that CST1 reduces cell apoptosis and promotes EMT and invasion, suggesting CST1 plays an important role in the progression and metastasis of breast cancer. Future studies are needed to uncover the underlying mechanisms of CST1 activity in breast cancer.

Collectively, our study is the first to clarify that CST1 could promote cell proliferation and clone formation, migration, and invasion in breast cancer cells. Importantly, upregulation of CST1 was correlated with a poor prognosis. Thus, CST1 may serve as a novel prognostic biomarker and a potential therapeutic target for breast cancer treatment.

## Electronic supplementary material

ESM 1 (PDF 440 kb)
