# Supplementary material for: Elevated expression of CST1 promotes breast cancer progression and predicts a poor prognosis
Source: J Mol Med (Berl). 2017 May 18;95(8):873–86. doi: 10.1007/s00109-017-1537-1 (PMC5515997; doi:10.1007/s00109-017-1537-1)
Supplement: Supplementary file 1 — (PDF 440 kb) [file 109_2017_1537_MOESM1_ESM.pdf]

Electronic supplementary material (ESM)

J Mol Med 2017

Title: **Elevated expression of CST1 promotes breast cancer progression and predicts a poor prognosis**

Da-nian Dai[1,2*],Yan Li[1*], Bo Chen[1,2*], Yong Du[1*], Shi-bing Li[1], Shi-xun Lu[3], Zhi-ping Zhao[4], Ai-jun Zhou[1], Ning Xue[1], Tian-liang Xia[1], Mu-sheng Zeng[1], Qian Zhong[1], Wei-dong Wei[1,2]

1.State Key Laboratory of Oncology in South China, Collaborative Innovation Center for Cancer Medicine, Sun Yat-Sen University Cancer Center, Guangzhou, Guangdong, China

2.Department of Breast Oncology, Sun Yat-Sen University Cancer Center, Guangzhou, China

3.Department of Pathology, Sun Yat-Sen University Cancer Center, Guangzhou, China

4.Institute of Hepatopancreatobiliary Surgery, Southwest Hospital, Third Military Medical University, Chongqing, China

*These authors contributed equally to this work

Corresponding authors:

Weidong Wei

weiwd@sysucc.org.cn

Tel.020-87343806

Qian Zhong

zhongqian@sysucc.org.cn

Tel.020-87342662

Supplementary tables

Table S1. Correlation between CST1 expression and clinicopathological variables of breast cancer cases

Variables	Cases (n=244)	CST1 expression		P value
		Low	High	
Age (years)				0.493
≤50	130	25 (19.2%)	105 (80.8%)	
>50	114	26 (22.8%)	88 (77.2%)	
Gender				
female	244	51 (20.9%)	193 (79.1%)	
Menarche age				**0.017***
≤16	200	36 (18.0%)	164 (82.0%)	
>16	44	15 (34.1%)	29 (65.9%)	
Menopause				0.675
no	145	29 (20.0%)	116 (80.0%)	
yes	99	22 (22.2%)	77 (77.8%)	
Tumor Size				**0.005***
≤2.5	84	26 (31.0%)	58 (69.0%)	
>2.5	160	25 (15.6%)	135 (84.4%)	
Pathological LN infiltrated				0.093

no	68	19 (27.9%)	49 (72.1%)	
yes	176	32 (18.2%)	144 (81.8%)	
Tumor location				0.358
left	124	23 (18.5%)	101 (81.5%)	
right	120	28 (23.3%)	92 (76.7%)	
ER status				0.580
negative	129	25 (19.5%)	103 (80.5%)	
positive	115	26 (22.4%)	90 (77.6%)	
PR status				0.103
negative	144	25 (17.4%)	119 (82.6%)	
positive	100	26 (26.0%)	74 (74.0%)	
Her-2 status				**0.045***
negative	168	41（24.4%）	127（75.6%）	
positive	76	10（13.2%）	65（86.8%）	
TNBC				0.380
negative	223	48（21.6%）	174（78.4%）	
positive	21	3（13.6%)	19(86.4%)	
Histological grade				**<0.001***
well differentiated (G1)	15	13 (86.7%)	2 (13.3%)	
moderately differentiated (G2)	143	31 (21.7%)	112 (78.3%)	
poorly differentiated (G3)	86	7 (8.1%)	79 (91.0%)	
Pathological Tumor status (pT)				0.484
T1	28	9 (32.1%)	19 (67.9%)	
T2	119	23 (19.3%)	96 (80.7%)	
T3	34	7 (20.6%)	27 (79.4%)	
T4	63	12 (19.0%)	51 (81.0%)	
Pathological TNM Staging				0.090
I-II	90	24 (26.7%)	66 (73.3%)	

III	154	27(17.5%)	127(82.5%)

* P < 0.05, statistically significant.

Abbreviation: ER: estrogen receptor, PR: progesterone receptor, T: tumor, N: node, TNM: tumor node metastasis , TNBC: triple negative breast cancer, OS: overall survival, DFS: disease-free survival, NR: not reached

Table S2. CST1 expression in breast cancer patients by Kaplan-Meier survival analysis (log-rank test)

Variable	Case	DFS (months)			OS (months)		
		Mean	Median	P-value	Mean	Median	P-value
Total							
low expression	51.0	127.7	NR	**<0.001***	134.5	NR	**<0.001***
high expression	193.0	82.1	82.7		83.8	84.7	
Tumor size diameter ≤ 2.5(cm)							
low expression	26.0	129.9	NR	**0.005***	138.0	NR	**0.002***
high expression	58.0	85.0	82.2		85.6	82.2	
Tumor size diameter > 2.5(cm)							
low expression	26.0	123.2	NR	**0.003***	126.9	NR	**0.002***
high expression	135.0	79.1	82.7		81.4	84.7	
Tumor differentiation							
T1-2							
low expression	32.0	119.2	NR	**0.031***	127.7	NR	**0.019***
high expression	115.0	88.0	83.2		87.8	93.0	
T3-4							
low expression	19.0	138.7	NR	**<0.001***	140.1	NR	**<0.001***
high expression	78.0	71.7	55.4		74.5	58.5	
Lymph node metastasis status							
Lymph node metastasis negative							
low expression	19.0	131.6	NR	0.171	131.6	NR	0.208
high expression	49.0	85.3	NR		102.2	NR	
Lymph node metastasis positive							
low expression	32.0	122.4	NR	**<0.001***	130.8	NR	**0.001***
high expression	144.0	72.7	55.0		74.6	58.5	

ER negative							
low expression	25.0	122.5	NR	**0.002***	130.7	NR	**<0.001***
high expression	103.0	71.1	47.1		74.0	47.8	
ER positive							
low expression	26.0	129.9	NR	**0.003***	130.8	NR	**0.003***
high expression	90.0	94.6	NR		94.7	NR	
PR negative							
low expression	25.0	126.4	NR	**0.001***	134.0	NR	**0.003***
high expression	119.0	74.1	58.5		75.6	59.7	
PR positive							
low expression	26.0	126.8	NR	**0.011***	127.8	NR	**0.012***
high expression	74.0	94.6	NR		96.9	NR	
HER2 negative							
low expression	41.0	130.8	NR	**<0.001***	139.3	NR	**<0.001***
high expression	127.0	78.9	82.2		80.4	82.7	
Her-2 positive							
low expression	10.0	103.4	NR	0.313	113.0	NR	0.177
high expression	66.0	82.3	NR		85.3	NR	
TNM staging							
I-II							
low expression	24.0	80.8	NR	0.202	132.1	NR	0.204
high expression	66.0	81.1	NR		95.6	NR	
III							
low expression	27.0	127.0	NR	**<0.001***	134.7	NR	**<0.001***
high expression	127.0	70.0	52.2		73.3	55.4	
Histologic grade							
G2-3							
low expression	39.0	94.4	NR	**<0.001***	95.3	NR	**<0.001***
high expression	191.0	81.6	NR		83.4	NR	

* P < 0.05, statistically significant.

Abbreviation: ER: estrogen receptor, PR: progesterone receptor, T: tumor, N: node, TNM: tumor-node-metastasis, OS: overall survival, DFS: disease-free survival, NR: not reached

Table S3. Univariate COX regression analysis for overall survival and disease-free survival in patients with breast cancer

	Overall survival			Disease-free survival		
Variables	HR	(95%CI)	p value	HR	(95%CI)	p value
Age (years) (≤50 vs >50)	0.891	0.598-1.326	0.568	0.889	0.597-1.325	0.564
Tumor location (right/left)	1.051	0.707-1.562	0.804	1.055	0.710-1.567	0.792
Tumor Size（cm）(≤2.5 vs >2.5)	1.680	1.073-2.632	**0.023***	1.619	1.033-2.537	**0.035***
Menarche age (years) (≤16 vs >16)	0.733	0.416-1.292	0.283	0.705	0.400-1.242	0.226
Menopause (no vs yes)	0.900	0.598-1.355	0.613	0.918	0.610-1.384	0.684
Histological grade (G3/G2/G1)	2.544	1.766-3.663	**<0.001***	2.380	1.647-3.438	**<0.001***
Tumor status (T4/T3/T2/T1)	1.209	0.997-1.465	0.054	1.209	0.995-1.468	0.056
Nodal status (N>0/N0)	4.544	2.286-9.033	**<0.001***	4.803	2.417-9.547	**<0.001***
TNM staging (I-II/III)	2.826	1.708-4.677	**<0.001***	2.931	1.772-4.850	**<0.001***
ER (negative vs positive)	0.514	0.341-0.776	**0.002***	0.485	0.321-0.732	**0.001***
PR (negative vs positive)	0.536	0.349-0.824	**0.004***	0.512	0.333-0.787	**0.002***
Her2 (negative vs positive)	1.363	0.900-2.064	0.143	1.449	0.958-2.193	0.079
TNBC (negative vs positive)	1.788	0.977-3.273	0.059	1.677	0.916-3.069	0.094
CST1 expression (High/Low)	5.611	2.443-12.888	**<0.001***	5.083	2.219-11.644	**<0.001***

* P < 0.05, statistically significant.

Abbreviation: CST1: cystatin SN, ER: estrogen receptor, PR: progesterone receptor, TNBC: triple negative breast cancer, OS: overall survival, DFS: disease-free survival, NS: not significant, HR: hazard ratio, 95%CI: 95% confidential interval

Table S4. Multivariate COX regression analysis for overall survival and disease-free survival in patients with breast cancer

	Overall survival			Disease-free survival		
**Variables**	**HR**	**(95%CI)**	**p value**	**HR**	**(95%CI)**	**p value**
Tumor Size (cm) (≤2.5 vs >2.5)	1.009	0.628-1.622	0.970	0.964	0.600-1.550	0.881
Histological grade (G3/G2/G1)	1.908	1.277-2.852	**0.002***	1.828	1.220-2.739	**0.003***
Nodal status (N>0/N0)	3.287	1.536-7.033	**0.002***	3.332	1.564-7.096	**0.002***
TNM staging (I-II/III)	1.647	0.924-2.938	0.091	1.739	0.978-3.090	0.059
ER (negative vs positive)	0.644	0.409-1.015	0.058	0.636	0.402-1.005	0.053
PR (negative vs positive)	0.752	0.470-1.203	0.235	0.751	0.469-1.204	0.235
CST1 expression (high/low)	3.390	1.428-8.052	**0.006***	2.971	1.258-7.015	**0.013***

* P < 0.05, statistically significant.

Abbreviation: CST1: cystatin SN, ER: estrogen receptor, PR: progesterone receptor, OS: overall survival, DFS: disease-free survival, NS: not significant, HR: hazard ratio, 95%CI: 95% confidential interval

Supplementary figures

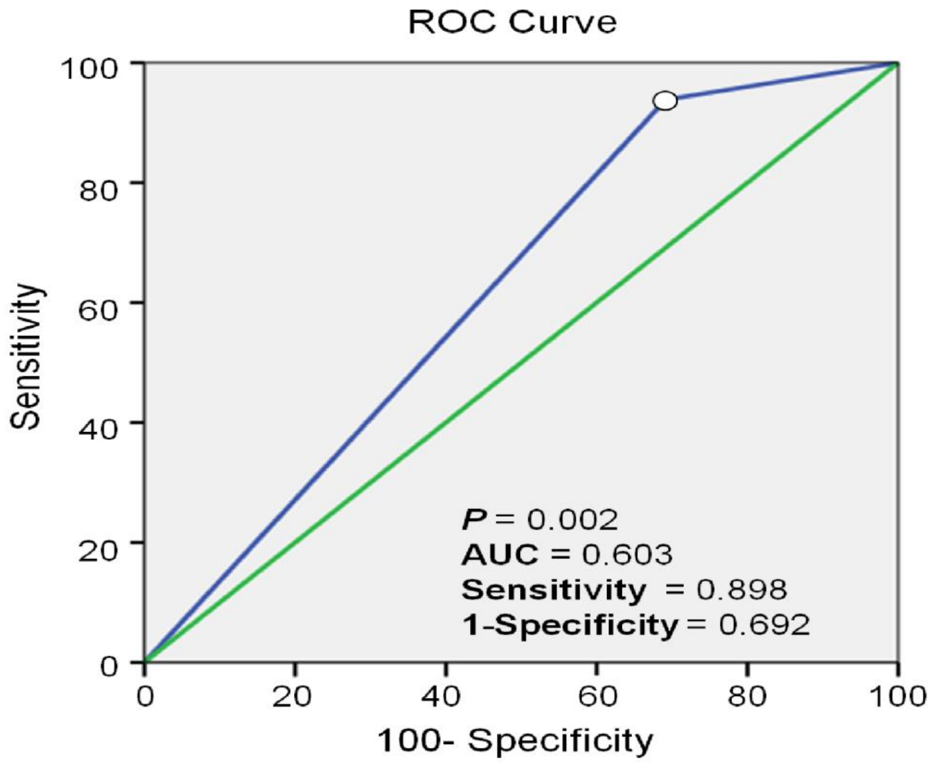

Fig. S1 Receiver operating curve (ROC) for the CST1 expression cutoff value plotted by survival status

The green line represents the completely random prediction. The blue line (the ROC curve) represents the ability of CST1 upregulation to predict survival status. The white dot represents the optimal prediction point.
